# Orthothanasia: science’s contribution to a dignified death

**DOI:** 10.17843/rpmesp.2024.414.13937

**Published:** 2024-11-12

**Authors:** Fernando M. Runzer-Colmenares, José Fonseca, Carolina Pérez-Agüero, José F. Parodi

**Affiliations:** 1 CHANGE Research Working Group, Carrera de Medicina Humana, Universidad Científica del Sur, Lima, Perú. Universidad Científica del Sur CHANGE Research Working Group Carrera de Medicina Humana Universidad Científica del Sur Lima Peru; 2 Society of Geriatrics and Gerontology of Peru, Lima, Peru. Society of Geriatrics and Gerontology of Peru Lima Peru; 3 “Cirujano Mayor Santiago Távara” Naval Medical Center, Callao, Peru. “Cirujano Mayor Santiago Távara” Naval Medical Center Callao Peru; 4 Peruvian Society of Geriatrics, Lima, Peru. Peruvian Society of Geriatrics Lima Peru; 5 Center for Aging Research (CIEN), Universidad de San Martín de Porres, School of Human Medicine, Lima, Peru. Universidad de San Martín de Porres Center for Aging Research (CIEN) Universidad de San Martín de Porres School of Human Medicine Lima Peru

To the editor. Palliative care (PC) is an approach to medical care and focuses on improving the quality of life of patients as well as their social and family environment in the face of serious or life-threatening illness. This type of care usually seeks to alleviate suffering through early identification of symptomatology by means of comprehensive palliative assessment and thus to provide interventions that mitigate pain and other psychosocial or spiritual challenges. PC should be provided to any type of person, regardless of age, diagnosis, prognosis, or socioeconomic status, and can even be provided, to some extent, along with curative treatments. Care at these stages is usually focused on the comfort of the patient and family, and is given higher priority than curative treatments or diagnostic efforts. Thus, PC starts before the disease with which the person is living reaches its terminal stage. People who have the opportunity of being early diagnosed with a disease that may reach the terminal stage have the option of discussing with their physician their preferences under these circumstances, and they do not end with the death of the patient, but include subsequent accompaniment of the family ^1^.

The authors focused on an exhaustive review of the literature regarding definitions in consensus, technical guidelines or clinical practice guidelines, summarizing the most important terminology in its most updated versions. The aforementioned search was carried out in Spanish, Portuguese and English. In addition to using databases such as PubMed/Medline, Embase, Scopus, Cochrane, Web of Science or Google Scholar, we also accessed documents from palliative care societies that had consensus published on their own web pages or on governmental pages (supplementary material). This synthesized information can be found in Table 1, and can be considered as the reference nomenclature for appropriate decision making in cases of need for PC. This letter aims to describe the current relevant terminology related to PC and to contextualize a current case related to the lack of legislation on the subject.

**Table 1:** Definition of important terms in palliative care and terminal illness.

Term	Definition
Palliative care	An approach that improves the quality of life of patients with advanced disease and their families by relieving suffering through early identification, impeccable assessment and treatment of pain and other physical, psychosocial and spiritual problems. This type of care considers death as a natural process and focuses on neither hastening nor delaying its outcome, providing support to live as actively as possible until death.
Euthanasia	Euthanasia is defined as the action of intentionally hastening the death of a terminally ill individual who consents to this act, with the aim of avoiding suffering. This definition focuses on the autonomy of the individual and his or her right to decide about his or her own life in circumstances of pain and terminal illness. The term “euthanasia” comes from Greek “eu” (good) and “thanatos” (death), reflecting the notion of a “good death”. However, the understanding and practice of euthanasia has varied throughout history and among different cultures, leading to ongoing debates about its morality and legality.
Dysthanasia	A phenomenon associated with the development of biomedicine and defined as the extension of the dying process of terminally ill patients by means of treatments that postpone the moment of death, without taking into account the patient’s quality of life. It is a term used in medicine to describe a “bad death” that occurs when the biological life of a terminally ill patient is extended by technological means without taking into account the patient’s quality of life. It is a practice that seeks to extend the life of terminally ill patients, but subjects them to much suffering, pain and anguish. Dysthanasia is also known as “therapeutic stubbornness” or “therapeutic striving”.
Orthothanasia	Death at its natural time, without medical intervention that unnecessarily extends life or hastens death. The natural and inevitable process of dying that respects a person’s right to die with dignity and is supported by palliative care. The word orthothanasia comes from Greek, ortho, meaning “right”, and thanatos, meaning “death”. It means not artificially extending the process of dying beyond what would be the natural process.
End-of-life care	It refers to the comprehensive care offered to patients in the terminal phase of their life, with the aim of reducing their suffering and increasing their quality of life, including psychological aspects and family support.
Assisted suicide	Intentionally assisting someone to end his or her life, usually with the help of a medical professional. It may involve people who are not terminally ill, but the term generally refers to medically assisted suicide, which is an end-of-life measure for a person suffering from a painful terminal illness.
Advance planning	It is the process of planning for future medical care with the goal of helping patients receive medical care that is aligned with their preferences, particularly in the context of serious illness or when approaching the end of life.
Anticipated will	Instructions a person gives to his or her physician or health care team about what kind of medical care he or she wishes to receive at the end of life. These instructions are given in advance, when the person is terminally ill or anticipating this situation.

Definitions adapted from “Palliative Care-Current Practice and Future Perspectives” ^7^.

Diseases such as cancer, dementia and other neurodegenerative diseases, liver cirrhosis, chronic kidney disease, chronic heart failure, pulmonary fibrosis, among others, have advanced stages as described by the literature, therefore the specialists involved in their management should build, as part of their medical residency, the competencies of pharmacological and non-pharmacological end-of-life management, including bioethical issues and especially basic definitions (Table 1). The need for community-based PC is also described, so that content and skills in this regard should be part of the curricula of the undergraduate programs of health professionals ^2^^,^^3^.

There is a shortage of postgraduate programs in Peru, whether medical or interprofessional, related to advanced disease and PC. There is still no medical residency program in palliative care. In this regard, the Regulation of Law No. 30012 was enacted in 2017, which establishes the guidelines for the implementation of the “National Palliative Care Plan for Oncologic and Non-Oncologic Diseases”, which was published in 2021 and has been being executed with discrete tangible indicators to date ^4^.

We highlight a well-known case of a woman under 50 years of age diagnosed with a degenerative autoimmune disease that progressively disabled her. The patient and her family, together with the Ombudsman’s Office, took her case to court (2019), therefore, the Peruvian Judicial Branch (2021) ordered the Ministry of Health and the Social Health Insurance (EsSalud) not to apply the Penal Code for the punishment of pious homicide, and thus end the patient’s life through euthanasia. This decision was ratified in July 2022 by the Supreme Court. In January of this year, EsSalud accepted the lifting of observations of the assisted death protocol, allowing the patient and her family to choose the health professional they trusted to perform the procedure, which was carried out in April of this year. In our opinion, the health system was able to provide palliative management based on orthothanasia in a timely manner, and thus the need to fight for a dignified death would not have been necessary.

Considering the legal implications of acts related to euthanasia and assisted suicide, it is imperative that the medical practice of PC and terminal illness (TI) be duly legislated, and there have to be protocols and clinical practice guidelines for its proper practice, always based on scientific evidence and the participation of duly accredited ethics committees. Patients with advanced diseases have known progression; frequently, the abrupt decline in their functionality and quality of life is caused by exacerbations that may be related to their chronic diseases, linked or not to the underlying advanced disease and sometimes caused by acute complications of an infectious or metabolic nature. Depending on the person’s condition, these situations are an opportunity for PC to move to end-of-life care. For example, it is not recommended to place an enteral feeding tube when a person with terminal dementia stops eating. Avoiding dysthanasia allows a dignified death, with less suffering, respecting the anticipated will, talking with the family, the patient and their environment, thus avoiding making decisions that entrap us in a legislation that is still in an incipient stage ^5^.

The right to health should be universal, and access to PC should not be an exception, as described in documents of the Catholic and Jewish Churches. For this reason, it is imperative to optimize the political and regulatory framework, the adequacy of services, the training of human resources and an information and research system that allows for monitoring and management decisions based on scientific evidence. The development and implementation of a long-term care system in Peru would be a fundamental framework for the optimization of the integral, coordinated and integrated implementation of the care needs of Peruvians, which are not only the responsibility of the health sector ^6^.

## References

[1] Knaul FM, Bhadelia A, Rodriguez NM, Arreola-Ornelas H, Zimmermann C (2018). The Lancet Commission on Palliative Care and Pain Relief-findings, recommendations, and future directions. Lancet Glob Health.

[2] Harrison KL, Boyd N, Ritchie CS (2023). Toward Gerineuropalliative Care for Patients with Dementia. N Engl J Med.

[3] Gonzalez-Smith J, Salzberg C, Smith M, Saunders R, McClellan M, Kahn CH (2023). Advancing the Future of "Care Without an Address": Recommendations from International Health Care Leaders.

[4] Aliaga Chávez RA, Bustamante Coronado RI, Chumbes Sipan ML, Grados Sánchez OA, Paredes Pascual RR, Salinas Paz JL (2023). Current state and future perspectives for palliative care in Latin America with a special focus on Peru. Future Oncol Lond Engl.

[5] Zheng R, Guo Q, Chen Z, Zeng Y (2023). Dignity therapy, psycho-spiritual well-being and quality of life in the terminally ill systematic review and meta-analysis. BMJ Support Palliat Care. septiembre de.

[6] Runzer-Colmenares FM, Parodi JF, Pérez Agüero C, Echegaray K, Samame JC (2019). Las personas con enfermedad terminal y la necesidad de cuidados paliativos una deuda pendiente de los servicios de salud. Acta Medica Peru.

[7] Bollig G, Zelko E (2024). Palliative Care - Current Practice and Future Perspectives.

