# Supplementary material for: Orthothanasia: science’s contribution to a dignified death
Source: Rev Peru Med Exp Salud Publica. 2024 Nov 12;41(4):445–7. doi: 10.17843/rpmesp.2024.414.13937 (PMC11797577; doi:10.17843/rpmesp.2024.414.13937)
Supplement: Supplementary material. — Available in the electronic version of the RPMESP. [file rpmesp-41-04-13937-s001.docx]

Material suplementario

PubMed/MEDLINE:

("Terminal Care"[Mesh] OR "Terminally Ill"[Mesh] OR terminal illness*[tiab] OR end-stage disease*[tiab] OR end of life[tiab])

AND

("Palliative Care"[Mesh] OR "Hospice Care"[Mesh] OR palliative[tiab] OR hospice[tiab] OR end-of-life care[tiab])

Embase:

('terminal disease'/exp OR 'terminally ill patient'/exp OR 'terminal illness':ti,ab OR 'end-stage disease':ti,ab OR 'end of life':ti,ab)

AND

('palliative therapy'/exp OR 'hospice care'/exp OR palliative:ti,ab OR hospice:ti,ab OR 'end-of-life care':ti,ab)

Scopus:

TITLE-ABS-KEY("terminal illness" OR "end-stage disease" OR "end of life")

AND

TITLE-ABS-KEY("palliative care" OR "hospice care" OR palliative OR hospice OR "end-of-life care")

Cochrane Library:

(MeSH descriptor: [Terminal Care] explode all trees OR MeSH descriptor: [Terminally Ill] explode all trees OR "terminal illness":ti,ab,kw OR "end-stage disease":ti,ab,kw OR "end of life":ti,ab,kw)

AND

(MeSH descriptor: [Palliative Care] explode all trees OR MeSH descriptor: [Hospice Care] explode all trees OR palliative:ti,ab,kw OR hospice:ti,ab,kw OR "end-of-life care":ti,ab,kw)

Web of Science:

TS=("terminal illness" OR "end-stage disease" OR "end of life")

AND

TS=("palliative care" OR "hospice care" OR palliative OR hospice OR "end-of-life care")

Google Académico:

("enfermedad terminal" OR "enfermedad en etapa terminal" OR "final de la vida")

AND

("cuidados paliativos" OR "cuidados de hospicio" OR paliativo OR hospicio OR "atención al final de la vida")
