# Supplementary material for: Orthothanasia: science’s contribution to a dignified death
Source: Rev Peru Med Exp Salud Publica. 2024 Nov 12;41(4):445–7. doi: 10.17843/rpmesp.2024.414.13937 (PMC11797577; doi:10.17843/rpmesp.2024.414.13937)
Supplement: Translation

# Translation: Ortotanasia: el aporte de la ciencia a una muerte digna

**Authors:** Fernando M. Runzer-Colmenares, José Fonseca, Carolina Pérez-Agüero, José F. Parodi

**Affiliations:** 1 CHANGE Research Working Group, Human Medicine Career, Universidad Científica del Sur, Lima, Peru.; 2 Sociedad de Geriatría y Gerontología del Perú, Lima, Perú.; 3 Centro Médico Naval «Cirujano Mayor Santiago Távara», Callao, Perú.; 4 Sociedad Peruana de Geriatría, Lima, Perú.; 5 Centro de Investigación del Envejecimiento (CIEN), Universidad de San Martín de Porres, Facultad de Medicina Humana, Lima, Perú.

Sr. Editor. Los cuidados paliativos (CP) son un enfoque de atención médica y se centran en mejorar la calidad de vida de los pacientes y el entorno sociofamiliar frente a enfermedades graves o potencialmente mortales. Este tipo de atención suele buscar el alivio del sufrimiento a través de la identificación precoz de sintomatología mediante la valoración paliativa integral y así poder realizar intervenciones que mitiguen el dolor y demás retos de índole psicosocial o espiritual. Los CP deberían ser brindados en cualquier tipo de persona, independientemente de la edad, diagnóstico, pronóstico, o nivel socioeconómico, incluso pueden proporcionarse, hasta cierto punto, de manera concomitante con tratamientos curativos. La atención en estas etapas suele centrarse en la comodidad o confort del paciente y su familia y, dándole mayor prioridad a ello que a tratamientos curativos o esfuerzos diagnósticos. Así, los CP inician antes de que la enfermedad con la que vive la persona llegue a su etapa de terminalidad. Las personas que tienen la oportunidad de un diagnóstico precoz de una enfermedad que puede llegar a una situación de terminalidad, tienen la opción de conversar con su médico sobre sus preferencias en esas circunstancias e, inclusive, no acaban con el fallecimiento del paciente, sino que incluyen un acompañamiento posterior a la familia ^1^.

Los autores nos enfocamos en realizar una revisión exhaustiva de la literatura a nivel de definiciones en consensos, guías técnicas o guías de práctica clínica, resumiendo la terminología más importante en sus versiones más actualizadas. La mencionada búsqueda fue realizada en español, portugués e inglés. Además de utilizar bases de datos como PubMed/Medline, Embase, Scopus, Cochrane, Web of Science o Google Académico, ingresamos también a documentos de sociedades de cuidados paliativos que tenían consensos publicados en sus propias páginas web o en páginas gubernamentales (material suplementario). Esa información sintetizada puede observarse en la tabla 1, y puede considerarse como la nomenclatura de referencia para la adecuada toma de decisiones en casos de necesidad de CP. El objetivo de la presente carta científica es describir la terminología actual pertinente relacionada con CP y contextualizar un caso actual relacionado con la falta de legislación sobre el tema.

**Tabla 1:** Definición de términos importantes en temática sobre Cuidados paliativos y enfermedad terminal.

Término	Definición
Cuidados paliativos	Enfoque que mejora la calidad de vida de los pacientes con enfermedad avanzada y sus familias, a través del alivio del sufrimiento mediante la identificación temprana, la evaluación impecable y el tratamiento del dolor y otros problemas, físicos, psicosociales y espirituales. Este tipo de cuidado considera la muerte como un proceso natural y se enfoca en no acelerar ni retrasar su desenlace, proporcionando soporte para vivir tan activamente como sea posible hasta la muerte.
Eutanasia	La eutanasia se define como la acción de acelerar intencionalmente la muerte de un individuo desahuciado que consiente este acto, con el objetivo de evitar su sufrimiento. Esta definición se centra en la autonomía del individuo y su derecho a decidir sobre su propia vida en circunstancias de dolor y enfermedad terminal. El término «eutanasia” proviene de las raíces griegas «eu» (bueno) y «thanatos» (muerte), lo que refleja la noción de una «buena muerte”. Sin embargo, la comprensión y la práctica de la eutanasia han variado a lo largo de la historia y entre diferentes culturas, lo que ha llevado a debates continuos sobre su moralidad y legalidad.
Distanasia	Fenómeno asociado al desarrollo de la biomedicina y se define como la prolongación del proceso de muerte de pacientes terminales mediante tratamientos que posponen el momento del fallecimiento, sin tener en cuenta la calidad de vida del paciente. Es un término utilizado en medicina para describir una «mala muerte” que ocurre cuando la vida biológica de un paciente terminal se prolonga mediante medios tecnológicos sin tener en cuenta su calidad de vida. Es una práctica que busca extender la vida de los pacientes terminales, pero los somete a mucho sufrimiento, dolor y angustia. La distanasia también es conocida como «obstinación terapéutica» o «encarnizamiento terapéutico».
Ortotanasia	Muerte en su momento natural, sin intervención médica que prolongue la vida innecesariamente ni aceleración de la muerte. El proceso natural e inevitable de la muerte que respeta el derecho de una persona a morir con dignidad y está respaldado por cuidados los paliativos. La palabra ortotanasia proviene de las palabras griegas orto, que significa «correcto», y thanatos, que significa «muerte». Significa no prolongar artificialmente el proceso de la muerte más allá de lo que sería el proceso natural.
Cuidados del final de la vida	Se refiere a la atención integral que se ofrece a los pacientes en la fase terminal de su vida, con el objetivo de disminuir su sufrimiento y aumentar su calidad de vida, incluyendo aspectos psicológicos y de apoyo a la familia.
Suicidio asistido	Asistir intencionalmente a alguien a terminar con su vida, generalmente con la ayuda de un profesional médico. Puede involucrar a personas que no están en fase terminal, pero el término generalmente se refiere al suicidio médicamente asistido, que es una medida de final de vida para una persona que sufre una enfermedad terminal dolorosa.
Planificación anticipada	Es el proceso de planificación de la atención médica futura con el objetivo de ayudar a los pacientes a recibir atención médica que esté alineada con sus preferencias, especialmente en el contexto de enfermedad grave o cuando se acerca el final de la vida.
Voluntades anticipadas	Instrucciones que una persona da a su médico o equipo asistencial sobre qué tipo de atención médica desea recibir al final de su vida. Estas instrucciones se dan de manera anticipada, cuando la persona se encuentra en una situación de enfermedad terminal o previendo esta situación.

Definiciones adaptadas de «Palliative Care-Current Practice and Future Perspectives» ^7^.

Enfermedades como el cáncer, la demencia y otras neurodegenerativas, cirrosis hepática, enfermedad renal crónica, falla cardíaca crónica, fibrosis pulmonar, entre otras, tienen etapas avanzadas descritas por la literatura, por lo que los médicos especialistas involucrados en su manejo deberían construir, como parte de su residentado médico, las competencias del manejo farmacológico y no farmacológico del final de la vida de cada una de ellas, incluyendo temas de bioética y en especial definiciones básicas (tabla 1). También se describen la necesidad de CP de base comunitaria, por lo que contenidos y capacidades al respecto deben formar parte de la currícula de los programas de pregrado de los profesionales de salud ^2^^,^^3^.

En el Perú existe una escasez de programas de posgrado, ya sean médicos o interprofesionales relacionados a enfermedad avanzada y CP. Aún no existe un programa de residencia médica en cuidados paliativos. Al respecto, en 2017 se promulgó el Reglamento de la Ley N° 30012, que establece las pautas para la aplicación del «Plan Nacional de Cuidados Paliativos para Enfermedades Oncológicas y no Oncológicas», que fue publicado en el año 2021 y viene siendo ejecutado con discretos indicadores tangibles a la fecha ^4^.

Resaltamos un caso mediático de una mujer de menos de 50 años con diagnóstico de una enfermedad degenerativa de carácter autoinmune que progresivamente la discapacitó. La paciente y su entorno llevaron (2019), de la mano de la Defensoría del Pueblo, su caso a los tribunales, siendo así que el Poder Judicial de Perú (2021) dio la orden al Ministerio de Salud y al Seguro Social de Salud (EsSalud) que no se aplique el Código Penal de sanción de homicidio piadoso, y así culminar con la vida de la paciente mediante eutanasia. Dicha decisión fue ratificada en julio del 2022 por la Corte Suprema. En enero del presente año, EsSalud aceptó el levantamiento de observaciones del protocolo de muerte asistida, permitiendo que la paciente y su entorno escogieran al profesional de salud de confianza para aplicar el procedimiento que fue realizado en abril de este año. En nuestra opinión, el sistema de salud pudo dar un manejo paliativo, basado en ortotanasia de forma oportuna, y así la necesidad de luchar por un fallecimiento digno no hubiese sido necesario.

Considerando las implicancias legales que tienen los actos relacionados con eutanasia y suicidio asistido, es imperativo que la práctica médica de CP y enfermedad terminal (ET) esté debidamente legislada, y que se cuente con protocolos y guías de práctica clínica para su ejercicio adecuado, siempre basándonos en la evidencia científica y la participación de comités de ética debidamente acreditados. Los pacientes con enfermedades avanzadas tienen trayectorias de evolución ya descritos; con frecuencia, el declive brusco en su funcionalidad y calidad de vida se produce por reagudizaciones que pueden estar relacionadas a sus enfermedades crónicas, vinculadas o no a la enfermedad avanzada de fondo y a veces provocadas por complicaciones agudas de carácter infeccioso o metabólico. Dependiendo del estado de la persona, estas situaciones son una oportunidad para que los CP pasen a ser cuidados del final de la vida. Por ejemplo, en una persona con demencia terminal que deja de comer, no está recomendado colocar una sonda de alimentación enteral. Evitar la distanasia permite un fallecimiento digno, con menos sufrimiento, respetando voluntades anticipadas, conversando con la familia, el paciente y el entorno, evitando así tomar decisiones que nos entrampen en un entorno de legislaciones que aún están en una etapa incipiente ^5^.

El derecho a la salud debe ser universal, y el acceso a los CP no debería ser una excepción, tal y como describen documentos de la Iglesia católica y judía. Por tal motivo es imperativa la optimización del marco político y normativo, la adecuación de los servicios, la capacitación del recurso humano y un sistema de información e investigación que permita el monitoreo y la toma de decisiones de gestión basada en evidencia científica. El desarrollo e implementación de un sistema de cuidados de largo plazo en Perú sería un marco fundamental para la optimización de la implementación integral, coordinada e integrada con las necesidades de cuidado de los peruanos, que no solamente son responsabilidad del sector salud ^6^.
